# The *Drosophila* Homologue of the Amyloid Precursor Protein Is a Conserved Modulator of Wnt PCP Signaling

**DOI:** 10.1371/journal.pbio.1001562

**Published:** 2013-05-14

**Authors:** Alessia Soldano, Zeynep Okray, Pavlina Janovska, Kateřina Tmejová, Elodie Reynaud, Annelies Claeys, Jiekun Yan, Zeynep Kalender Atak, Bart De Strooper, Jean-Maurice Dura, Vítězslav Bryja, Bassem A. Hassan

**Affiliations:** 1 VIB Center for the Biology of Disease, Leuven, Belgium; 2 Center for Human Genetics, University of Leuven School of Medicine, Leuven, Belgium; 3 Doctoral Program in Molecular and Developmental Genetics, University of Leuven Group Biomedicine, Leuven, Belgium; 4 Institute of Experimental Biology, Faculty of Science, Masaryk University, Brno, Czech Republic; 5 Institut de Génétique Humaine/Centre National de la Recherche Scientifique UPR1142, Montpellier, France; 6 Laboratoire Neurogénétique et Mémoire, Département Génétique et Développement, Montpellier, France; 7 Institute of Biophysics of the Academy of Sciences of the Czech Republic, Brno, Czech Republic; University of Zurich, Switzerland

## Abstract

Wnt Planar Cell Polarity (PCP) signaling is a universal regulator of polarity in epithelial cells, but it regulates axon outgrowth in neurons, suggesting the existence of axonal modulators of Wnt-PCP activity. The Amyloid precursor proteins (APPs) are intensely investigated because of their link to Alzheimer's disease (AD). APP's in vivo function in the brain and the mechanisms underlying it remain unclear and controversial. *Drosophila* possesses a single APP homologue called APP Like, or APPL. APPL is expressed in all neurons throughout development, but has no established function in neuronal development. We therefore investigated the role of *Drosophila* APPL during brain development. We find that APPL is involved in the development of the Mushroom Body αβ neurons and, in particular, is required cell-autonomously for the β-axons and non-cell autonomously for the α-axons growth. Moreover, we find that APPL is a modulator of the Wnt-PCP pathway required for axonal outgrowth, but not cell polarity. Molecularly, both human APP and fly APPL form complexes with PCP receptors, thus suggesting that APPs are part of the membrane protein complex upstream of PCP signaling. Moreover, we show that APPL regulates PCP pathway activation by modulating the phosphorylation of the Wnt adaptor protein Dishevelled (Dsh) by Abelson kinase (Abl). Taken together our data suggest that APPL is the first example of a modulator of the Wnt-PCP pathway specifically required for axon outgrowth.

## Abstract 2

The *Drosophila* homolog of the Alzheimer's disease protein APP, known as APPL, regulates axon growth during brain development.

## Author Summary

Wnt Planar Cell Polarity (PCP) signaling is a universal regulator of polarity in epithelial cells, but in neurons it regulates axon outgrowth, suggesting the existence of axonal modulators of Wnt-PCP activity. The Amyloid Precursor Proteins (APPs) are intensely investigated because of their link to Alzheimer's disease (AD). APP's in vivo function in the brain and the mechanisms underlying it remain unclear and controversial. In the present work we investigate the role of the *Drosophila* neuron-specific APP homologue, called APPL, during brain development. We find that APPL is required for the development of αβ neurons in the mushroom body, a structure critical for learning and memory. We find that APPL is a modulator of the Wnt-PCP pathway required for axonal outgrowth, but not for cell polarity. Molecularly, both human APP and fly APPL are found in membrane complexes with PCP receptors. Moreover, we show that APPL regulates PCP pathway activation through its downstream effector Abelson kinase (Abl), which modulates the phosphorylation of the Wnt adaptor protein Dishevelled (Dsh) and the subsequent activation of Wnt-PCP signaling. Taken together our data suggest that APPL is the first example of a neuron-specific modulator of the Wnt-PCP pathway.

## Introduction

The Wnt Planar Cell Polarity (PCP) pathway is a highly conserved regulator of cellular orientation within the plane of an epithelium [1],[2]. Genetic and molecular studies in *Drosophila* indicate Disheveled (Dsh), a cytoplasmic transducer of Wnt signaling; Frizzled (Fz), a seven-transmembrane receptor for Wnt ligands; and Van Gogh (Vang), a four-pass transmembrane protein, as core Wnt-PCP proteins. Intriguingly, the Wnt-PCP pathway regulates axon outgrowth rather than neuronal polarity during brain development of both vertebrates and *Drosophila* [3]–[5]. The Amyloid Precursor Protein (APP) is a member of a highly conserved family of type I transmembrane proteins that includes APP, APLP1, and APLP2 [6] in mammals and APP-Like, or APPL, in *Drosophila melanogaster* [7]. APP proteins show not only structural but also functional conservation, as exemplified by the ability of human APP to rescue behavioral phenotypes of APPL null flies [8]. APP is the subject of intense research because of genetic and biochemical links to Alzheimer's disease (AD), whereby the proteolytic processing of APP generates the Amyloid Beta peptide whose accumulation in the brain is widely thought to induce neurodegeneration [9]–[12]. Despite these efforts, the normal physiological function of APP in vivo in the nervous system remains elusive and highly controversial. This is due to the lack of a consensus over the neuronal phenotypes in null mutant animals and the mechanism of APP action in vivo. APP knock-out mice are viable and show developmental neuronal deficits, namely cortical migration and agenesis of the corpus callosum, at variable penetrance depending on genetic background [13]–[15]. In contrast, another in vivo report, based mainly on gain of function and RNA interference experiments, suggests that APP may be required for developmental axonal degeneration [16], although it is unclear whether APP knock-out mice show these phenotypes. Finally, initial findings proposed an axonal transport function for APP [17] but later studies strongly questioned the presence of these defects in APP knock-out mice [18].

Mechanistically, there is also disagreement on whether APP acts cell autonomously or non autonomously. For example, an extensive network of molecular interactions has been described for the intracellular domain of APP [19], yet a knock-in of APP lacking the intracellular domain appears to rescue physiological and learning deficits reported in APP knock-out mice, suggesting that the intracellular domain is dispensable [20]. Because current models based on Amyloid toxicity do not provide a complete explanation for the onset of neuronal dysfunction in AD, it has long been argued that greater attention needs to shift towards understanding the normal physiological function of APP in order to assess its potential contribution to AD pathology [21],[22]. Therefore, a mechanistic understanding of the in vivo physiological function of APP proteins is of paramount importance. To elucidate the function and mechanism of action of APP proteins in vivo, we first investigated *Drosophila* APPL, the APP homologue in the fruit fly, as a model system. We show that APPL is a novel neuronal-specific modulator of the PCP pathway required for the robustness of axonal outgrowth during the development of the Mushroom Bodies (MB), a *Drosophila* center for learning and memory. APPL carries out this function through facilitating the PCP-specific phosphorylation of the Wnt adaptor protein Dishevelled (Dsh/Dvl) by the Abelson kinase (Abl). Furthermore, we show that APPL is part of the membrane complex formed by Wnt-PCP core proteins. Finally, biochemical and cell biological analyses show that human APP immunoprecipitates mammalian PCP proteins and that APP proteins are necessary for Dvl phosphorylation in response to the PCP ligand Wnt5a. Therefore, the APP proteins represent a novel and conserved family of neuronal modulators of Wnt-PCP signaling required for the robustness of brain wiring during development.

## Results

### APPL Is a Robustness Factor Required Cell-Autonomously for MB β-Lobe Development

**Figure 1:**
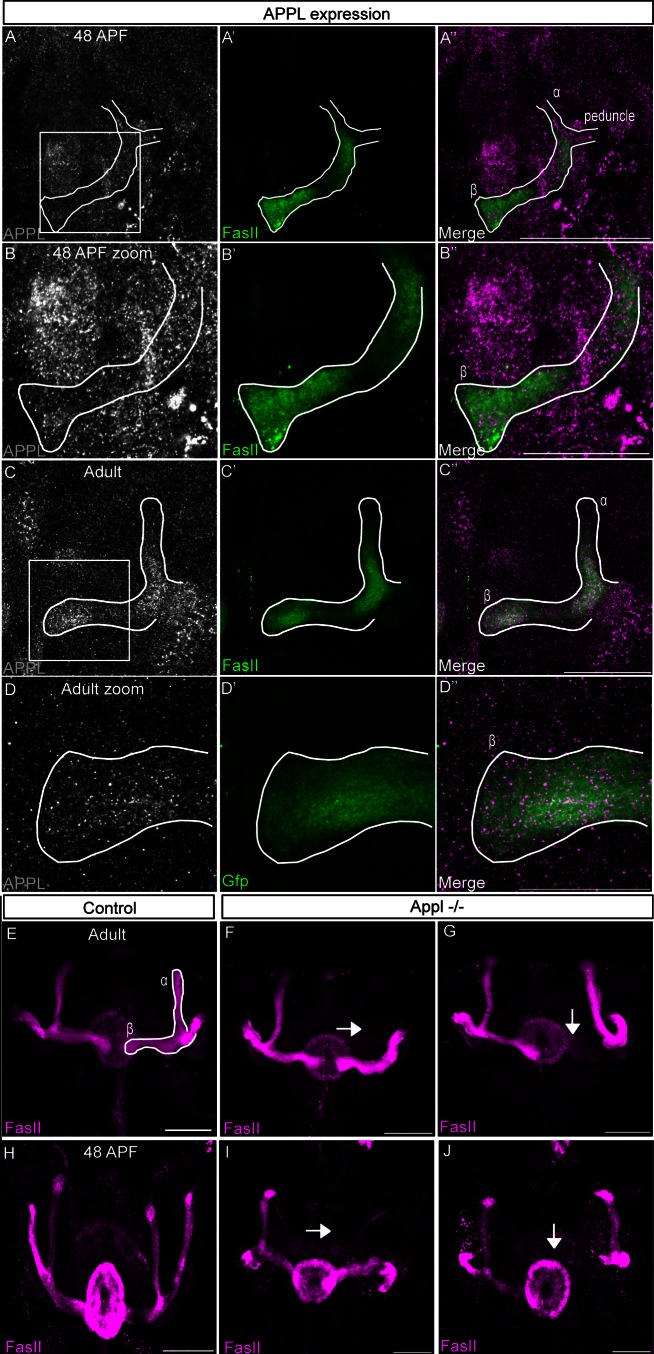
APPL is a robustness factor required for Mushroom Bodies development. (A–D) Developmental APPL expression in MB. Immuno-fluorescence analysis using anti-APP-Cterm (A–D in magenta) and anti-FasII (A′–D′ in green) antibodies. The A‴–D‴ panels show the merge of the FasII and GFP channel for the indicated samples. APPL is expressed at high levels in the developing brain and is enriched along MB axons both during development and in adult stages. (A, B) 48 h APF MB lobes. The images are single confocal stacks; zoom shows 63× magnification of the boxed area (scale bar, 50 µm in A, 25 µm in B). (C) Adult MB axons. The images are single confocal stacks (scale bar, 50 µm). (D) Zoom shows 63× magnification of the boxed area in panel C. The images are *z*-projections of two confocal image stacks (step size, 0.6 µm; scale bar, 25 µm). (E–G) Adult MB lobes labeled with FascilinII antibody (FasII). All images are *z*-projections of confocal image stacks (scale bar, 50 µm). (E) Morphologically normal α/β neurons in control adult brain. (F–G) Structure of α/β neurons in Appl^d^w* null mutant adult brains. In the absence of APPL, MB lobes show an aberrant pattern of growth in 26% of the analyzed sample (*n* = 101). In particular, in 14% of the cases (F), the α lobe fails to project towards the dorsal side of the brains (as indicated by the arrow), whereas in 12% of the cases (G), the β lobe fails to project towards the midline (as indicated by the arrow). (H) Morphologically normal α/β neurons of a 48APF Canton S brain. (I–J) Morphologically aberrant α/β neurons of an Appld w*48APF brain. The α- (I) and β-lobe (J) loss observed in the adult brain is already present at 48 APF, thus suggesting that is not due to degeneration but rather to failure in axon growth.

*Drosophila* APPL is a neuronal-specific protein expressed in most, if not all, neurons throughout development and adult life. In particular, APPL is highly expressed in the developing *Drosophila* MB, especially the so-called αβ neurons (Figure 1A–D). Flies null for *Appl* (henceforth *Appl* ^−/−^) are viable, fertile, and reported to show no gross structural defects in the brain [8]. While a requirement for APPL in learning and memory specifically in adult flies has been shown [23], the function of its pan-neuronal expression throughout development remains unknown, as does the in vivo mechanism of its action(s).

**Figure 2:**
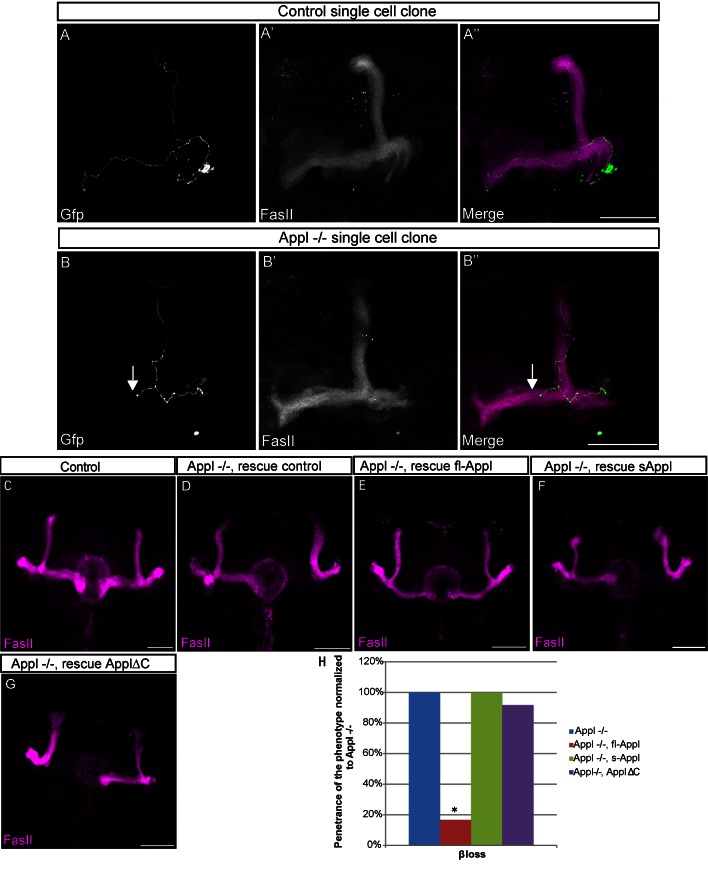
APPL is cell-autonomously required for β-lobe development. (A–B) *Z*-projections of confocal image stacks of GFP-labeled clones. Recombination was induced at 0–24 h APF. Immunofluorescence analysis of adult MBs using anti-GFP (green) and anti-FasII (A′, B′ in magenta) antibodies (scale bar, 50 µm). The A″ and B″ panels show the merge of the FasII and GFP channel for the indicated samples. (A) Morphologically normal α/β neurons in single-cell control clones obtained by crossing *FRT19A; ry^506^* flies with *FRT19A,tub-Gal80,hsFLP/FM7;UAS-CD8-GFP/CyO;OK107* (*n* = 41 clones). (B) *Appl^d^* single-cell clones. Clones were obtained by crossing *Appl^d^ FRT19A/FM7* with *FRT19A, tub-Gal80, hsFLP/FM7; UAS-CD8-GFP/CyO*; OK107. The cell-autonomous loss of APPL leads to β-lobe loss in 10% of the clones analyzed as indicated by the arrow (*n* = 44 clones). (C–G) Adult MB lobes labeled with FasII antibody. All the images are *z*-projections of confocal image stacks (scale bar, 50 µm). (C) Morphologically normal α/β neurons in a control adult brain. (D) Morphologically aberrant αβ neurons in Appl^d^w*/Y;L/+;P247Gal4 analyzed as a control for the rescue experiment (*n* = 47). In 13% of the brains the β axons failed to grow towards the midline. (E) Morphologically normal αβ neurons in Appl^d^w*/Y;UAS-Appl/+;P247Gal4 adult brains. The re-introduction of full-length APPL in MBs during development is sufficient to rescue the β-lobe defect. Only 2% of the samples show a phenotype; *p* value = 0.03467 calculated with G-test (*n* = 50). (F) Morphologically aberrant αβ neurons in Appl^d^w*/Y;UAS-sAppl/+;P247Gal4. The re-introduction of a secreted form of APPL fails to rescue the β-lobe defect, where 12% of the analyzed brains showed phenotype. *p* value = 0.9831 calculated with G-test (*n* = 50). (G) Morphologically aberrant αβ neurons in Appl^d^w*/Y;UAS-ApplΔC/+;P247Gal4. The re-introduction of a form of APPL lacking the C-terminal domain fails to rescue the β-lobe defect, where 11% of the analyzed brains showed phenotype (*n* = 54); *p* value = 0.8863 calculated with G-test. (H) The graph shows the penetrance of the β-lobe loss in the rescue flies normalized against the penetrance of the phenotype in the Appl^d^w* background. * Indicates a *p* value<0.05 calculated with G-test.

We began addressing the function of APPL in neuronal development by carefully examining the development of the *Drosophila* MB in *Appl* ^−/−^ mutant flies. The MB derives from two groups of four neuroblasts, one in each hemisphere, that sequentially generate three subsets of neurons: the γ-, α′β′ and αβ neurons, where APPL is highly expressed. Each αβ neuron projects an axon that branches into a dorsal “α branch” and a medial “β branch.” The fascicles generated by each of these branches are referred to as the α lobe and β lobe. The α and β lobes can be easily visualized using the anti-FascilinII (FasII) antibody. The lobes were present and morphologically normal in 97 adult control animals (Figure 1E) examined. In contrast, 26% of *Appl* ^−/−^ brains examined (*n* = 101) showed axonal defects (Figure S1). Specifically, 14% of the brains show α-lobe loss (Figure 1F), whereas 12% of the brains show β-lobe loss (Figure 1G). These defects are developmental in origin as they can be observed during αβ lobe formation at 48 h of pupal development (Figure 1H–J). To ascertain whether APPL acts cell autonomously to regulate MB axonal outgrowth, we generated GFP-marked single *Appl* ^−/−^ cell clones using the MARCM technique [24]. While none of the control clones showed any defects (Figure 2A), 10% of the mutant clones showed lack of β-lobe growth (Figures 2B and S2A), similar to the penetrance observed in null mutant brains. However, none of the mutant clones showed loss of α-lobe growth. Taken together, these data indicate that APPL is required for normal MB axonal outgrowth and that it is required cell-autonomously for the growth of the β-lobe and non-cell autonomously for the growth of α lobe. To verify the specificity of the *Appl* ^−/−^ phenotype, we rescued the defects by restoring APPL expression in αβ neurons. Expression of full-length membrane-bound APPL, but not a secreted form or a form lacking the intracellular domain (ApplΔC), strongly suppresses the β-lobe loss phenotype (Figures 2C–H and S2B). These results indicate that APPL is required as a full-length, membrane-tethered protein and that the intracellular signaling downstream of APPL is necessary for normal β-lobe outgrowth. Interestingly, secreted APPL strongly reduces the loss of the α lobe (Figure S2C and S2D), confirming that APPL acts non-autonomously in α-lobe outgrowth. All together, the results suggest that APPL is a robustness factor for an unknown axon growth signal whereby *Appl* ^−/−^ αβ neurons are at a phenotypic threshold that causes them to fail to grow in approximately 26% of the cases.

### Abelson Kinase Is a Downstream Effector of APPL Required for MB Axon Outgrowth

**Figure 3:**
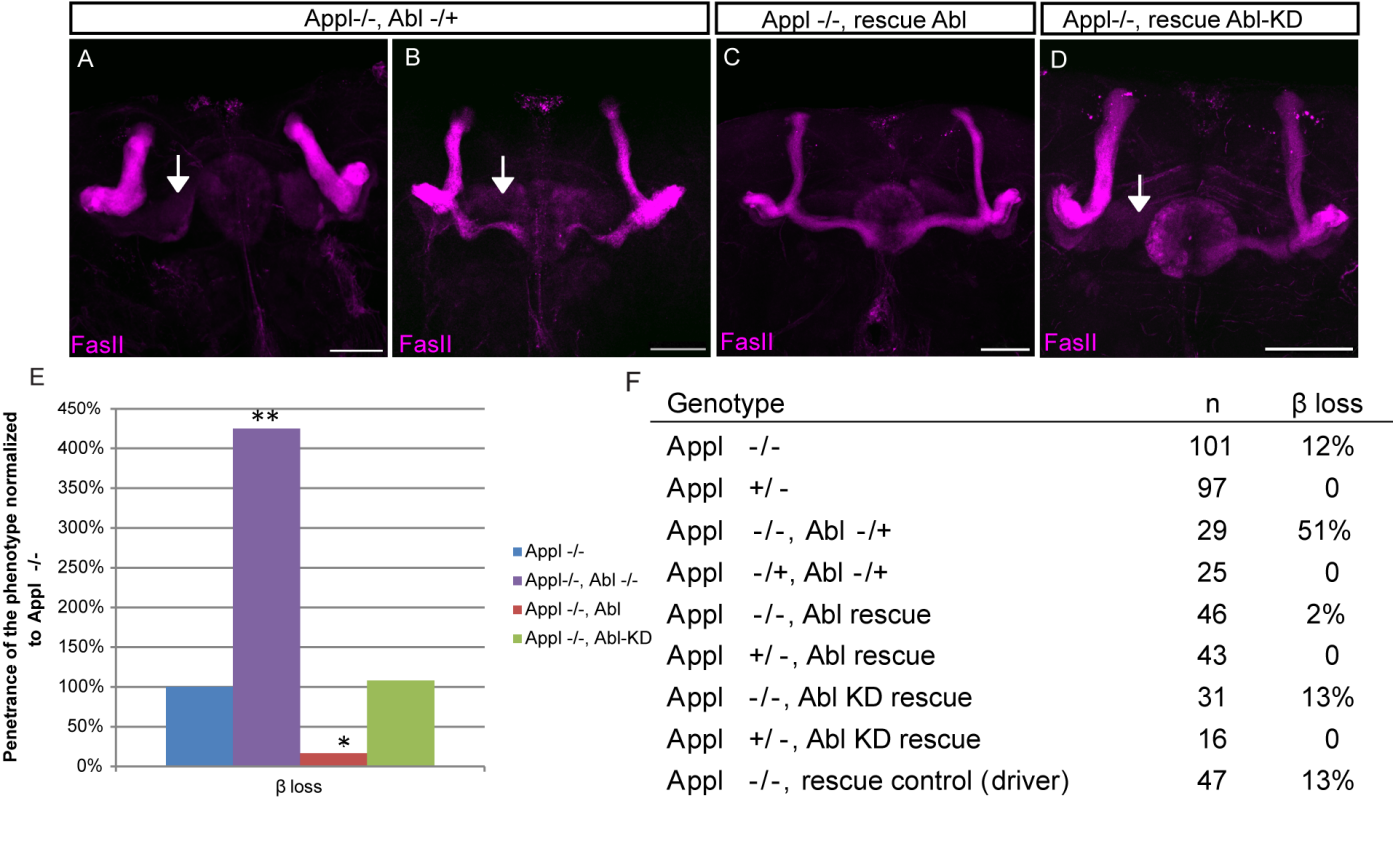
Abelson kinase is a downstream effector of APPL required for MB axons outgrowth. (A–B) Loss of β lobes in Appl^d^w*/Y;Abl^4^/+ adult brains. (A) In 40% of the flies that have lost one copy of Abl in the *Appl* ^−/−^ background, no axons grow toward the midline (indicated by the arrow), whereas in 10% of the cases (B) only few axons project normally (*n* = 29); *p* value = 1.29E-02 calculated with G-test. (C) Morphologically normal αβ neurons in Appl^d^w*/Y;UAS-Abl/+;P247Gal4 adult brains. Overexpression of Abl in the MB rescues the β-lobe loss in the *Appl* ^−/−^ background. Only 2% of the analyzed samples show β-lobe loss (*n* = 46); *p* value = 0.03192 calculated with G-test. (D) Morphologically aberrant αβ neurons in Appl^d^w*/Y;UAS-Abl-KD/+;P247Gal4 adult brains. Overexpression of a *Kinase dead* form of Abl fails to rescue the β-lobe loss in the *Appl* ^−/−^ background. Thirteen percent of the analyzed brains showed β-lobe loss as indicated by the arrow (*n* = 31). (E) The graph shows the penetrance of β-lobe loss in the Abl rescue flies and in the flies heterozygous for Abl, normalized against the penetrance observed in the Appl^d^w* background. (F) The table lists the number of brains analyzed in the rescue experiments. * Indicates a *p* value<0.05 calculated with G-test; ** indicates a *p* value<0.001 calculated with G-test.

To unravel the mechanism by which APPL supports MB axon outgrowth, we chose to focus on the cell-autonomous function of APPL in the β lobe. A previous study using APPL gain of function indicates that APPL overexpression induces axonal outgrowth that is dependent on Abl kinase activity [25]. We asked whether Abl kinase also acts downstream of APPL during MB β-lobe growth. First, we tested if APPL genetically interacts with Abl. To this end we analyzed the adult MB morphology of *Appl* ^−/−^; *Abl* ^−/+^ flies. Loss of one copy of Abelson causes a dramatic increase (up to 51%) in complete (41%) or partial (10%) β-lobe loss, compared to *Appl* ^−/−^ alone (Figure 3A, 3B and 3E, 3F). As controls we analyzed the siblings heterozygous for both *Appl* and *Abl* (*Appl* ^−/+^; *Abl* ^−/+^) and observed no phenotypes (Figure 3F). Similarly to *Appl* ^−/−^ mutants alone, the phenotypes arise at early developmental stages (Figure S3A). To further confirm that Abl is the downstream mediator of APPL signaling during β-lobe growth, we tested if overexpression of Abl specifically in MB αβ neurons rescues the *Appl* null phenotype. We find that wild-type Abl, but not a Kinase Dead (Abl-KD) form of Abl, rescues the *Appl* null phenotype (Figure 3C–F) to the same extent as MB αβ expression of APPL itself (Figure S2B). Taken together these data indicate that Abl is the effector of APPL required for the β-lobe growth. Next, we further characterized the downstream pathway involved.

### APPL Is a Novel Neuronal-Specific Modulator of PCP Signaling

**Figure 4:**
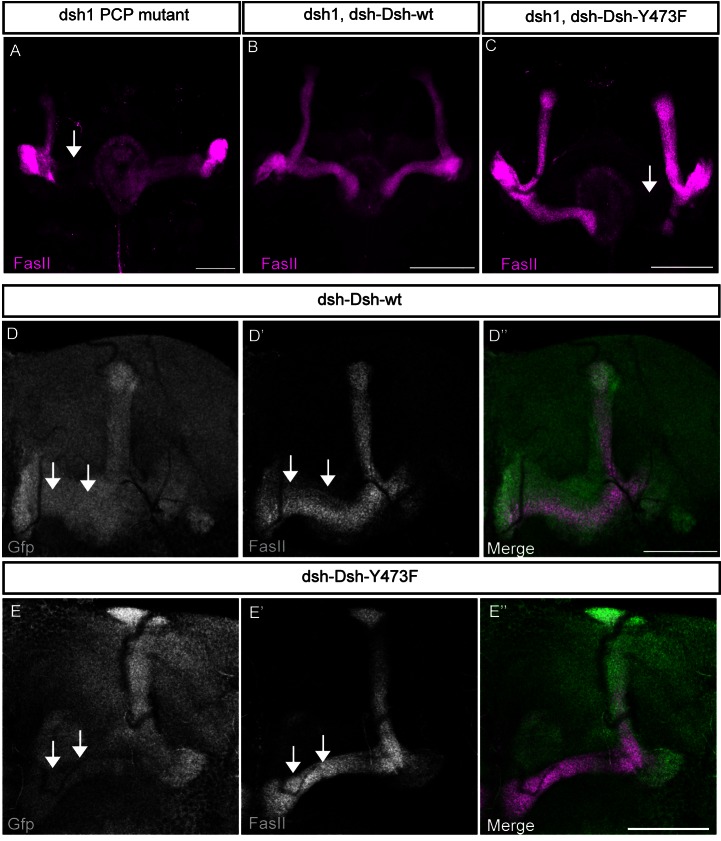
Dsh phosphorylation is required for MB β-lobe development. Structure of αβ neurons labeled with FasII antibody. All the images are *z*-projections of confocal image stacks (scale bar, 50 µm). (A) Morphologically aberrant αβ neurons in *dsh^1^* adult brains. Flies homozygous for a PCP specific allele of dsh (*dsh^1^*) show a phenotype comparable to *Appl* ^−/−^ mutants but with increased penetrance of β-lobe loss as indicated by the arrow (30%; *n* = 36). (B) Morphologically normal αβ neurons in *dsh^1^/Y*;*dsh-DshGFP-wt/+* adult brains. Reintroduction of wild-type Dsh in the *dsh^1^* mutant background completely rescues β-lobe loss with no brains showing defects (*n* = 31). (C) Morphologically aberrant αβ neurons in *dsh^1^/Y*;;*dsh-DshGFP-Y473F/+* adult brains. Reintroduction of a Tyrosine 473 phospho-mutant form of Dsh in a *dsh^1^* mutant background fails to rescue the β-lobe loss as indicated by the arrow (*n* = 41). (D–E) Expression pattern of Dsh-GFP in ;*dsh-DshGFP-wt/TM6b* and of Dsh-Y473F-GFP in ;*dsh-DshGFP-Y473F/+*. Immuno-fluorescence analysis of adult brains using anti-GFP (green) and anti-FasII (D′, E′ in magenta) antibodies (scale bar, 50 µm). All images are *z*-projections of two confocal sections (0.9 µm steps). The D″ and E″ panels show the merge of the FasII and GFP channel for the indicated samples.

It has been recently shown that Abl phosphorylates Disheveled (Dsh), a core intracellular component of the Wnt pathway, on the Tyrosine 473. This modification is required for the efficient activation of the PCP signaling pathway in epithelial cells [26]. Interestingly, in the nervous system the Wnt-PCP pathway is required for robust axonal outgrowth both in *Drosophila* [27] and mouse [28],[29]. More recently, several PCP pathway components, like the Wnt receptor Frizzled (Fz), Flamingo (Fmi), Strabismus (Stbm or Vang), and Dsh, have been shown to play a role in the correct targeting and bifurcation of MB axons [4],[30],[31]. Indeed, we observe that flies harboring a PCP-specific mutation in Dsh (*dsh^1^*) [32] show the same MB developmental defects observed in *Appl* ^−/−^ flies (Figure 4A).

Together, these observations prompted us to speculate that APPL acts to facilitate Wnt-PCP pathway activation during MB development by mediating Dsh phosphorylation by Abl. To verify this hypothesis we tested if the phosphorylation of Dsh on Y473 is required for MB development. We analyzed *dsh^1^* flies harboring one of two genomic rescue constructs: a wild-type construct (*dsh-DshGFP-wt*) or a Tyrosine 473 phospho-mutant construct (*dsh-DshGFP-Y473F*). Whereas the restoration of wild-type Dsh fully rescues the *dsh^1^* MB phenotype (Figures 4B and S4A), Dsh^Y473F^ completely fails to do so (Figures 4C and S4A). To exclude that Dsh^Y473F^ failure to rescue the phenotype is due to a perturbation in the expression pattern resulting from the point mutation, we performed anti-GFP staining on adult brains of both transgenic lines. As shown in Figure 4D and 4E, both wild type and mutant Dsh are expressed in the MB lobes. These results clearly indicate that Abl phosphorylation of Dsh on Tyrosine 473 and the subsequent activation of Wnt-PCP signaling are required for the β-lobe growth.

**Figure 5:**
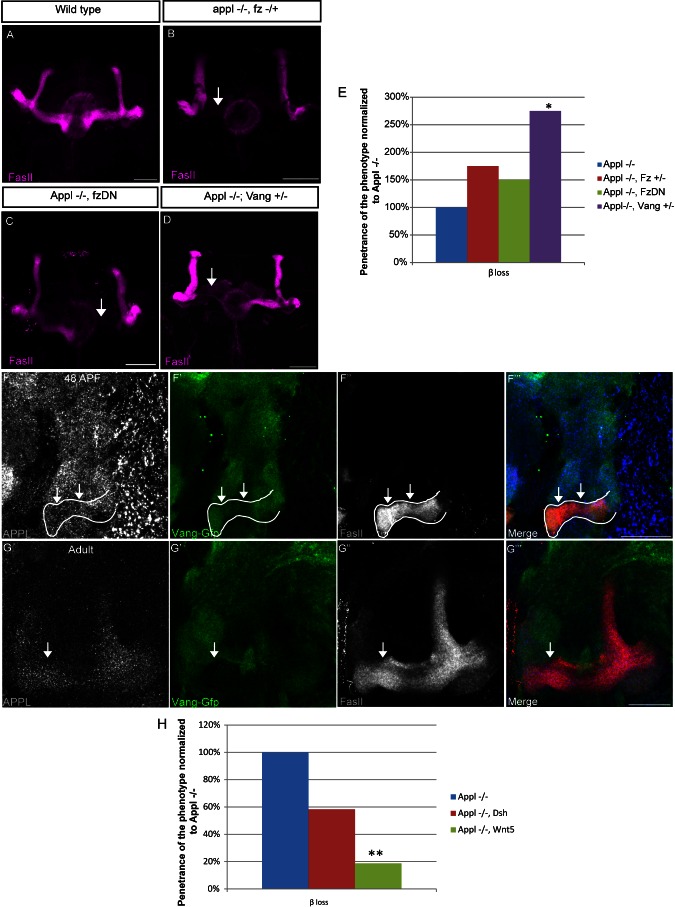
APPL interacts with PCP signaling during MB development. (A–D) Structure of αβ neurons labeled with the FasII antibody. All the images are *z*-projections of confocal image stacks (scale bar, 50 µm). (A) Morphologically normal αβ neurons in a control adult brain. (B) Morphologically aberrant αβ neurons in Appl^d^w*/Y;;Fz^KD^/+ adult brains. Loss of one copy of Fz in the *Appl* ^−/−^ background increases moderately, but not significantly, the penetrance of β-lobe loss (indicated by the arrow) to 21% (*n* = 28); *p* value = 0.2166 calculated with G-test. (C) Morphologically aberrant α/β neurons in Appl^d^ w*/Y;UAS-Fz-DN/+;P247Gal4/+ adult brains. Overexpression of a dominant negative form of Fz in the *Appl* ^−/−^ background increases moderately, but not significantly, the penetrance of the phenotype to 18% (*n* = 28); *p* value = 0.4226 calculated with G-test. (D) Morphologically aberrant αβ neurons in Appld w*/Y;Vang^stbm-6^/+ adult brains. Loss of one copy of Vang in the *Appl* ^−/−^ background increases the penetrance of the β-lobe loss up to 33% (*n* = 21); *p* value = 0.02304 calculated with G-test. (E) The graph shows the penetrance of β-lobe loss in genetic interaction experiments, normalized against the penetrance in the Appl^d^w* background. (F, G) APPL and Vang localization during development in brain of flies *Act-Stbm-EYFP* expressing an EYPF tagged form of Vang under the control of the Actin promoter. Immuno-fluorescence analysis using anti-APP-Cterm (F, G in blue), anti-GFP (F′, G′ in green), and anti-FasII (F″, G″ in red) antibodies. The images are single confocal stacks (scale bar, 25 µm). The F‴ and G‴ panels show the merge of the FasII and GFP channel for the indicated samples. (F) At 48 h APF, Vang is broadly expressed along the MB β axons, where also APPL is expressed as indicated by the arrow. (G) At adult stage, the level of Vang detectable in the β axons is strongly reduced compared to the previously analyzed time point, while APPL is enriched in the MB neurons (as indicated by the arrow). (H) The graph shows the penetrance of the β-lobe loss in the Appld^w*^ flies overexpressing Dsh or Wnt5 (*p* value equal to 0.1287 and 0.0006692, respectively). Activation of Wnt-PCP signaling upon Wnt5 signaling rescues the β-lobe phenotype. ** Indicates a *p* value<0.001 calculated with G-test.

Collectively, the data suggest the exciting possibility that APPL may be a neuronal-specific component of the Wnt-PCP pathway. To address this issue, we first asked if APPL interacts genetically with core members of the Wnt-PCP pathway like the classical Wnt-PCP receptor Fz and the canonical Wnt-PCP protein Van Gogh/Strabismus (Vang/Stbm). We first analyzed the β-lobe of *Appl* ^−/−^; *Fz* ^−/+^ flies. Reduction of Fz in the APPL null background increases the frequency of the β lobe loss up to 21%, whereas no phenotype is observed in control siblings (Figures 5B, E and S5A). This interaction is specific to the MB because expression of a dominant negative form of Fz (Fz-DN) in *Appl* ^−/−^ αβ neurons yields similar results (Figures 5C, 5E and S5A). In both experiments described, the increase in β-lobe loss is relatively mild compared to the dramatic increase in β-lobe loss due to APPL-Abl epistasis for example, suggesting that APPL and Fz may act together for Wnt-PCP activation. To clarify this, we tested if inhibition of Fz alone is sufficient to induce the β phenotype. Overexpression of Fz-DN in αβ neurons of wild-type flies did not cause any morphological defects (Figure S5A and S5B). These data were further confirmed by the analysis of *Fz* mutant αβ clones of different sizes. None of the analyzed MB clones showed morphologically aberrant axons (Figure S5C). To rule out compensation by Fz2, we examined Fz2 expression in the brain and found that it is not detectable in MB (unpublished data). Furthermore, MARCM clones null for Fz2 alone or Fz and Fz2 together show no aberrant morphology (Figure S5D and S5E). Therefore, APPL function is a critical determinant of the role of PCP in the outgrowth of MB β axons. To further ascertain the interaction with the Wnt-PCP pathway, we analyzed if APPL interacts with the Wnt-PCP four-pass transmembrane protein Vang/Stbm. Reduction of Van Gogh in the *Appl* null background (*Appl* ^−/−^;*vang* ^−/+^) increases the frequency of the β-lobe loss phenotype to 33% (Figures 5D,E and S5A), whereas no phenotype is observed in control siblings. APPL is also expressed in the developing fly retina (Figure S5F), where the PCP pathway regulates the polarity of photoreceptor cells. However, we did not observe defects in photoreceptor polarity (Figure S5G–I), suggesting that the role of APPL in Wnt-PCP signaling is specific to axonal outgrowth. Together, the data above identify APPL as the first neuronal-specific modulator of the Wnt-PCP pathway's role in axonal outgrowth. Next, we analyzed if the expression pattern of Vang and APPL overlaps during MB development. For this purpose, we used a line that expresses a YFP tagged form of Vang under the control of the Actin promoter. As shown in Figure 5F, during the development of the β lobe both APPL and Vang are expressed at a high level in the growing axons. Interestingly, in adult stage, APPL expression is reduced in the rest of the brain and enriched in the αβ neurons while Vang levels are strongly reduced in these axons (Figure 5G). Moreover, in the developing fly retina, APPL and Vang do not colocalize and are found in juxtaposed domains (Figure S5L). Taken together these results suggest that both APPL and Vang are present in developing αβ axons where their genetic interaction is required for the correct development of the β axons. On the contrary, the two proteins are expressed in different compartments in the fly retina where APPL function is not required for PCP activity.

Finally, to confirm that PCP signaling is indeed positively modulated by APPL and that the activation of the signaling is required for the β-lobe growth, we performed rescue experiments with Dsh. As shown in Figure 5F, overexpression of Dsh in the *Appl* ^−/−^ background strongly reduces (7%) the β-lobe loss but does not fully rescue the phenotype. This result indicates that the phosphorylation of Dsh by Abelson is the limiting step in the activation of PCP signaling; increasing the amount of Dsh present in the neurons improves the phenotype, but probably the endogenous Abelson is not sufficient to phosphorylate the whole pool of Dsh. To overcome this problem we decided to enhance the activation of PCP signaling by overexpressing Wnt5 in the *Appl* ^−/−^ background. Interestingly, overexpression of Wnt5 significantly rescues the β-lobe loss (Figure 5H), indicating that the PCP signaling activation is required for the development of the β lobe and is reduced in absence of APPL.

### APP Is Required for Dvl2 Phosphorylation in Response to Wnt5 and Forms Complexes with PCP Core Proteins

**Figure 6:**
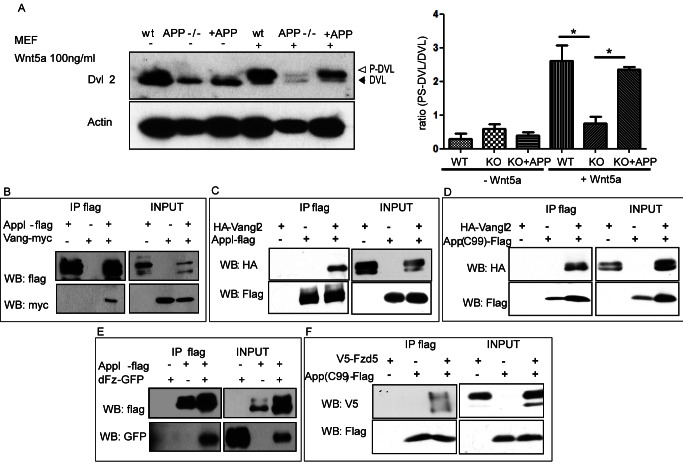
APP is required for the proper response to Wnt5 and forms complexes with PCP core proteins. (A) Analysis of the responsiveness of wt MEFs and MEFs lacking all the APP isoforms to Wnt5 treatment. MEFs were treated for 2 h with rmWnt5a and subsequently analyzed by Western blot. After the Wnt5 treatment, Dvl2 is phosphorylated and this modification is indicated by a shift of the band detected by Dvl2 Ab. KO MEFs respond less efficiently to Wnt5. Re-introduction of hAPP rescues the responsiveness to Wnt5. The graph shows a quantification of the ratio between phospho-Dvl2 and Dvl2 in the analyzed samples. * Indicates a *p* value<0.05 calculated with one-way ANOVA plus Tukey's multiple comparison test. (B) Co-immunoprecipitation (Co-IP) of Appl-FLAG and Vang-Myc. The tagged proteins were co-expressed in HEK293T cells and immunoprecipitated with anti-FLAG antibody. Vang-Myc can be precipitated upon IP of Appl-FLAG. (C) Co-IP of Appl-FLAG and human Vangl2-HA. Human Vangl2-HA can be precipitated upon IP of Appl-FLAG. (D) Co-IP of human APP (C99)-FLAG and human Vangl2-HA. Human Vangl2-HA can be precipitated upon IP of APP (C99)-FLAG, indicating that interaction with PCP proteins is a conserved feature of APP proteins. (E) Co-IP of Appl-FLAG and dFz-GFP. *Drosophila* Fz can be precipitated upon IP of Appl-FLAG. (F) Co-IP of human APP (C99)-FLAG and human V5-Fzd5. V5-Fzd5 can be precipitated upon IP of APP (C99)-FLAG.

The previously described results raise two important questions. First, is the interaction between APPL and the Wnt-PCP pathway conserved in mammalian APP proteins? Second, if so, do the genetic interactions observed in *Drosophila* reflect a biochemical association of APPL/APP with the Wnt-PCP receptors? To address these issues, we first investigated if mouse APP proteins mediate the phosphorylation of mouse Disheveled (Dvl) in response to the Wnt-PCP ligand Wnt5a. To this end, we analyzed Dvl phosphorylation in response to Wnt5a treatment in wild-type versus APP/APLP2 double knock-out mouse embryonic fibroblast (dKO MEFs). In WT MEFs, Dvl2 phosphorylation increases dramatically upon treatment with Wnt5a. In contrast, Dvl2 in dKO MEFs completely fails to respond to Wnt5a treatment (Figure 6A). The effect on the activation of Dvl2 is a direct consequence of APP loss because upon reintroduction of APP cDNA Dvl2 phosphorylation is restored (Figure 6A). Next, we tested if APPL and APP interacts with core Wnt-PCP receptor proteins. In particular, we performed co-immunoprecipitation (Co-IP) analyses of tagged proteins expressed in HEK-293T cells. As shown in Figures 6B and S6B, APPL immunoprecipitates Vang when the two proteins are co-expressed in the same cells. *Drosophila* APPL immunoprecipitates human Van Gogh 2 (Vangl2) (Figures 6C and S6C). Importantly, this multiprotein complex can only be detected when the proteins are expressed in the same cells, but not when lysates from separately expressing cells are mixed (Figure S6E). This observation also accounts for the absence of rescue observed when a form of APPL lacking the C terminal is expressed in the *Appl* ^−/−^ animals (Figure 2H). Similarly, the membrane tethered C-terminus of human APP (APP-C99) immunoprecipitates Vangl2 (Figures 6D and S6D). Moreover, we tested if APPL also immunoprecipitates other PCP receptors like Fz. As shown in Figures 6E and S6F, APPL immunoprecipitates Fz, suggesting that the core PCP proteins and APPL might form a multiprotein complex on the membrane, responsible for the efficient activation of the pathway. Similarly, the membrane-tethered C-terminus of human APP (APP-C99) immunoprecipitates human Fzd5 (Figure 6F).

## Discussion

AD is a neurodegenerative disorder characterized by progressive loss of neurons in specific regions of the brain that correlates with progressive impairment of higher cognitive functions. A growing body of evidence identifies the APP and its metabolite the Aβ peptide as main players in the pathogenesis of AD. In particular, the accumulation of Aβ peptides in the brain seems to be the trigger of the pathological cascade that eventually results in neuronal loss and degeneration [33]. Despite efforts to characterize the molecular mechanisms underlying Aβ's toxic function, it is still not clear what triggers the accumulation of the peptide and how this is correlated with the pathogenesis of the disease and the dementia. In fact, most of the work done to unveil the pathogenesis of the disease has focused on the analysis of Aβ-peptide and the search for its receptors and downstream effectors. Even though the numerous in vitro studies performed in cell culture identified several molecules that interact with Aβ peptide, the in vivo biological relevance of these interactions remains to be clarified. The amyloid cascade hypothesis has also dominated the search for AD treatments, but the promising molecular candidates developed to modulate the Aβ peptide and reached clinical trials failed [34],[35]. Finally, over the last few years many studies indicated that there is no linear correlation between the accumulation of the peptide and the cognitive decline, leading to a revision of the amyloidogenic hypothesis. Taken together, these observations suggest that the accumulation of the peptide is not the only cause of the pathology and that other factors are involved. Interestingly, under physiological conditions APP is mainly found in its uncleaved or α-cleaved form, suggesting that the shift towards amyloidogenic processing not only increases the production of Aβ peptide but also depletes the pool of APP that undergoes non-amyloidogenic processing, with hitherto unknown consequences. It is therefore of paramount importance to understand the physiological role of APP and how perturbing this role could contribute to the pathogenesis of the disease. An important contribution to the study of the function of a protein comes from the analysis of the knock-out (KO) animals. In the case of APP, several KO models have been generated and analyzed in detail both from the morphological and behavioral point of view [13],[36]–[38]. Despite these efforts, the normal physiological function of APP in vivo in the nervous system remains largely elusive and highly controversial. This is due to the lack of consensus over the neuronal phenotypes in null mutant animals and the mechanism of action in vivo. The data collected by different labs confirmed the involvement of APPs in development and function of the nervous system, but these studies do not provide an in-depth analysis of the development of the brain during the pre-natal stages or the molecular mechanism underlying APPs' putative functions. We therefore took advantage of *Drosophila melanogaster* to further analyze the consequence of loss of APP Like (APPL) during brain development.

In the present study we demonstrate that APPL is involved in brain development of *Drosophila melanogaster*, particularly in the Mushroom Body (MB) neurons. We show that APPL is required for the development of αβ neurons. In *Appl* ^−/−^ flies, MB neurons fail to project the α lobe in 14% of the cases and the β-lobe in 12% of the cases (Figure 1). Further analysis of the phenotype reveals that APPL is required cell-autonomously for the development of the β lobe and non-cell autonomously for the development of the α lobe. In fact, single cell *Appl* ^−/−^ clones display only β-lobe loss and no α loss. The re-introduction of a full-length, membrane-tethered form of APPL, but not a soluble form, rescues β-lobe loss (Figure 2). This is of particular interest because it confirms that, similar to mammalian APPs, the physiological role of APPL is mediated both by its full-length form, required in the neurons to achieve the correct β-lobe pattern, and by its soluble form (sAPPL) that regulates the extension of the α lobe. Moreover, the rescue data indicate that, at least in this context, the function of sAPPL is mediated not by homo-dimerization with the full-length form but by some other receptor, hitherto unknown. Further experiments are required to clarify the sAPPL non-cell autonomous effect, but we hypothesize that it might be involved in modulating signaling mediated by the cells that surround the MB axons. Taken together, the analysis of the *Appl* ^−/−^ animals confirmed the important role of APPs during brain development but reinforced the idea that the phenotypes are present with incomplete penetrance and might be subtle. It would therefore be of interest to analyze the phenotype of the KO mice in greater detail and, in particular, to better characterize the APP's downstream pathway leading to these defects.

Moreover, the results described clearly support a model of APPL as a novel, neuronal-specific positive modulator of the Wnt-PCP pathway (Figure 4). The PCP pathway was initially described because of its role in tissue polarity establishment and, in particular, of its regulation of cell orientation in plane of an epithelium. Among the different processes regulated by PCP signaling, we are interested in axon growth and guidance. It has been described that mice null for *Fzd3*/*Ceslr3* ^−/−^ genes show severe defects in several major axon tracts like thalamocortical, corticothalamic, and nigrostriatal tracts, defects of the anterior commissure, and similarly to APP KO mice, the variable loss of the corpus callosum [5],[39].

The molecular mechanism underlying the function of PCP-signaling in regulating tissue polarity has been broadly studied. The current model suggests that, upon polarized expression of the different core proteins, Dsh is recruited to the membrane via Fz and leads to the activation of a cascade of small GTPases finally resulting in cytoskeleton rearrangements. In the case of regulation of axon growth and guidance, it is less clear how the signaling is regulated and transmitted to the cytoskeleton. A recent publication suggested that during axon growth the transmembrane PCP receptor-like Vang and Fzd are localized at the growth cone area on the tip of the fillopodia, thus suggesting that in this context the asymmetric localization is not needed [28].

**Figure 7:**
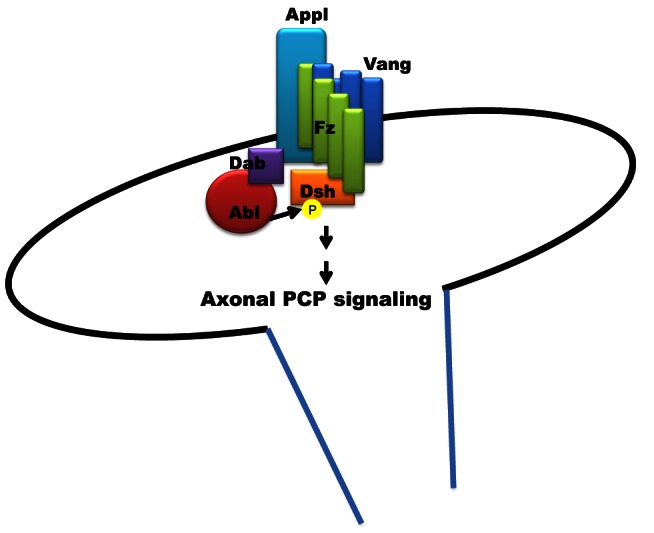
APPL is a novel modulator of Wnt-PCP signaling required for axon guidance. Schematic representation of the proposed model. APPL is a novel regulator of Wnt-PCP pathway. In the presence of Wnt signaling, Fz binds Dsh, which needs to be phosphorylated by Abelson kinase to correctly relocalize to the membrane. We propose that Appl is part of the membrane complex formed by the core PCP proteins Fz1 and Vang. In turn, Appl recruits Abelson kinase to the complex and positively modulates Dsh phosphorylation. The subsequent activation of the signaling is required for MB β-axon growth.

Furthermore, Dsh needs to relocalize from the cytoplasm to the membrane to ensure the proper activation of PCP signaling, and this is dependent on its phosphorylation status. Singh and colleagues showed that Abelson is one kinase responsible for this modification, but the receptor upstream of the kinase was not identified [26]. Based on the evidence we generated, we propose that APPL is a novel regulator of Wnt-PCP pathway involved in axon growth and guidance (Figure 7). This is of interest because while the PCP core proteins are ubiquitously expressed, APPL is restricted to the nervous system, suggesting that it could be the first described tissue-specific modulator of the pathway.

Mechanistically, we propose that APPL-Abl complex modulates Dsh via dual protein-protein interactions. First, Abl might have an intrinsic affinity for its substrate Dsh [26]. Secondly, this interaction is strengthened or stabilized by the inclusion of APPL in a PCP receptor complex. This dual affinity complex leads to increased PCP signaling efficiency at the developing growth cone. Both biochemical and physiological data show that this function is highly conserved in mammalian APP, suggesting that it may play a similar role in the mammalian brain. The canonical-Wnt signaling pathway has already been connected to AD pathogenesis because of its link to the tau-kinase GSK-3β. Interestingly, no clear link between the Wnt-PCP pathway and this neurodegenerative disorder has been made. Previous reports [27],[28] show that, in flies and mice, Jun N-terminal Kinase (JNK) is the final effector of PCP in axon outgrowth and JNK was shown to be required for the effect of APP overexpression in the fly [25],[40]. Interestingly, JNK signaling has also been linked to the neuronal loss observed in AD [41]. It is therefore worth investigating whether the physiological function of APP as a neuronal PCP modulator explains the JNK-AD connection.

## Materials and Methods

### Fly Stocks

Drosophila stocks used include Appl^d^ w^*^, Appl^d^,FRT19A w^*^, elav^C155^,hsFLP,w*;UAS-mCD8::GFP.,UAS-lacZ/CyO;tubP-GAL80,FRT2A/TM6,Tb,Hu, hsFlp,UAS-CD8-GFP;;FRT2A,tubGal80/TM3;OK107, hsFlp, UAS-CD8-GFP; FRT2A, tubGal80/TM3;OK107, Flp122; sp/CyO;Fz p21,ri,FRT2A/TM2, fz2^C1^ri,FRT2A/TM3,Sb, yw,hsflip;Fz1^H51^Fz2^C1^ri^FRT2A^/TM2, UAS-Fz-DN/CyO;P247Gal4/TM6c, UAS-Abl-KD/CyO;P247Gal. Abl^4^ kar^1^ red^1^ e^1^/TM6B, Tb^1^, w*;UAS-Abl/CyO;P247, w; fz ^[KD]^/TM3,Sb, Vang^stbm-6^, w;201Y,UAS-GFP, FRT19A;ry^50^, FRT19A,tub-Gal80,hsFLP/FM7;UAS-CD8-GFP/CyO;OK107, UAS-Appl/CyO;P247Gal4, UAS-sAppl/Cyo;P247Gal4, w^1^, dsh^:1^, dsh>Dsh-GFP (J7)/TM6. dshV26,dsh>Dsh-GFP; dsh>Dsh-GFP Y473F, and Act-stbm-EYFP/TM3.

### Accession Numbers/ID Numbers

APPL (FBgn0000108), fz (FBgn0001085), fz2 (FBgn0016797), Fzd5 (NP_003459.2), dsh (FBgn0000499), dvl1 (AAB65242.1), dvl2 (AAB65243.1), Vang (FBgn0015838), Vagl2 (NP_065068.1), and Abl (FBgn0000017).

### Immunochemistry

Larval, pupal, or adult brains were dissected in phosphate buffered saline (PBS) and fixed in 3.7% formaldehyde in PBT (PBS+ Triton ×100 0.1%) for 15 min. The samples were subsequently rinsed three times in PBT and blocked in PAX-DG for 1 h. Following these steps, the brains were incubated with the primary antibody diluted in PAX-DG overnight at 4°C. This incubation was followed by three washes with PBT and a subsequent incubation with the appropriate fluorescent secondary antibodies for 2 h at RT. After three rinses in PBT, the brains were put in 50% Glycerol diluted in PBS and then mounted in Vectashield (Vector Labs) mounting medium. The following antibodies were used: rabbit anti-GFP (Invitrogen, 1∶1,000), mouse anti-FasII (Hybridoma Bank, 1∶50), rabbit anti-APP-C-term (kind gift of Bart de Strooper lab, 1∶5,000), and anti-Phalloidin TRITC (1∶1,000).

### Microscopy and Image Analysis

The mounted brains were imaged either on a LEICA DM 6000 CS microscope coupled to a LEICA CTR 6500 confocal system or on a Nikon A1-R confocal (Nikon) mounted on a Nikon Ti-2000 inverted microscope (Nikon) and equipped with 405, 488, 561, and 639 nm lasers from Melles Griot. The pictures were then processed using ImageJ and Adobe Photoshop.

### MARCM Procedure

Crosses were set up at 25°C and transferred every day. We transferred 0 to 24 pupae in a fresh vial, and they were heath shocked for 45′ at 37°C and shifted back at 25°C until eclosion. The morphology of the MB clones was analyzed in flies 0–7 d old.

### Cell Culture and Treatments

WT MEFs, APP/APLP2 double KO MEFs, APP/APLP2 double KO+hAPP, and HEK-293T cells were propagated in DMEM, 10% FCS, 2 mM L-glutamine, 50 units/ml penicillin, 50 units/ml streptomycin. MEFs (200,000 cells per well) were seeded in 24-well plates for biochemical analyses. MEFs were treated 2 d after seeding with rmWnt5a (R&D Systems) for 2 h. Cells were harvested for immunoblotting by direct lysis in 1× Laemmli buffer followed by boiling at 95°C for 5 min. Control stimulations were done with 0.1% BSA in PBS.

### Gel Electrophoresis and Western Blots

Protein from total cell lysates/samples was resolved in 10% polyacrylamide gels (SDS-PAGE) under denaturing conditions and then transferred to nitrocellulose membranes. The blots were probed using polyclonal anti-FLAG M2 (F1804, Sigma-Aldrich 1∶1,000), monoclonal anti-Myc (M4439,Sigma-Aldrich 1∶1,000), anti-Dvl1 (sc-8025, Santa Cruz Biotechnologies, 1∶1,000), anti-Dvl2 (#3224, Cell Signaling Technologies, 1∶1,000), anti-V5 (R960-25, Invitrogen, 1∶1,000), anti-HA (HA.11, MMS-101R, Covance, 1∶2,000), and anti-beta-actin (sc-1615, Santa Cruz Biotechnology, 1∶2,000). Bands were visualized using anti-IgG HRP-conjugated secondary antibodies, and the ECL Western Blotting Detection System (GE Healthcare, UK).

### Co-Immunoprecipitation

For the Co-IP of Drosophila proteins, pCDNA3-APPL-FLAG and pCDNA3-Vang-Myc were transiently transfected in HEK293T cells (4.5×10^6^ cells per 10 cm dish) using Fugene HD (Roche). After 3 d, cells were collected in Lysis Buffer (150 mM NaCl, 50 mM Tris/HCl pH 7.5, 10% glycerol, 0.4% Nonidet P-40) and cleared with Dynabeads M-270 epoxy (Invitrogen) for 45′ at 4°C. After the clearing, lysates (half volume) were incubated with anti-FLAG covalently conjugated to Dynabeads M-270 (pre-saturated with BSA) for 1 h at 4°C. Beads were then washed, and bound proteins were resuspended in 6× Laemmli and subjected to SDS-PAGE followed by Western blot analysis. For the Co-IP of Drosophila and human proteins, HEK293 cells grown at 50% confluency on 10-cm plates were transfected with 6 µg of each plasmid. After 2 d, cells were lysed for 15 min in 1 ml of lysis buffer ([0 mM Tris buffer pH 7.4, 150 mM NaCl, 1 mM EDTA, 0.5% NP40 supplemented with 1 mM DTT and protease inhibitor cocktail (Roche, cat. no. 11836145001)]. Lysates were centrifuged at 13,200× g for 20 min at 4°C, supernatants were collected, and 0.4 ml of the supernatant was incubated with 1 µg of indicated immunoprecipitating antibody for 1 h at 4°C. Immunoprecipitates were collected on Protein G sepharose beads by overnight rotation, washed four times with lysis buffer, resuspended in 2× Laemmli sample buffer, and subjected to SDS-PAGE followed by Western blot analysis. The antibodies used for immunoprecipitation include FLAG M2 (F1804 Sigma-Aldrich), anti-HA (ab9110; Abcam), anti-Myc (M4439, Sigma-Aldrich), and anti-V5 (R960-25, Invitrogen).

## Supporting Information

Figure S1 Appl is required during the development of Mushroom Bodies for α- and β-lobe growth. (A) The table shows the number of brains analyzed to characterize the Appl loss of function phenotype.
(PDF)

Figure S2 APPL is required cell-autonomously for the β-lobe outgrowth. (A)The table lists the number of brains analyzed in the MARCM experiments. (B, C) The table lists the number of brains analyzed in the rescue experiments. (D) Adult MB lobes labeled with FascilinII II antibody (FasII). The image is a *z*-projection of confocal image stacks (scale bar, 50 µm). Morphologically normal αβ neurons in Appl^d^w*/Y;UAS-sAppl/+;P247Gal4 adult brains. The reintroduction of soluble APPL in MBs during development strongly reduces the loss of the α lobe.
(PDF)

Figure S3 Abelson kinase is the downstream effector of APPL in MB development. (A) Structure of α/β neurons of a Appld;;Abl4 48APF brain. The image is a *z*-projection of confocal image stacks (scale bar, 50 µm). The MBs are labeled with anti-FasII antibody. β-lobe loss is detectable already at 48 APF, similarly to what is observed in the *Appl* ^−/−^ background.
(PDF)

Figure S4 Dsh phosphorylation is required for MB development. (A) The table lists the number of brains analyzed in the *dsh* rescue experiments.
(PDF)

Figure S5 Appl interacts with the PCP signaling during MB development. (A) The table lists the number of brains analyzed in the PCP genetic interaction experiments. (B) Structure of α/β neurons labeled with anti-FasII antibody. The image is a *z*-projection of confocal image stacks (scale bar, 50 µm). Morphologically normal α/β neurons in UAS-FzDN/+;P247Gal4/+ adult brains. Expression of a dominant negative form of fz in the MB is not sufficient to induce defects. (C–E) *Z*-projections of confocal image stacks of GFP-labeled MARCM clones. Immuno-fluorescence analysis of adult MB, using anti-GFP (green) and anti-FasII (magenta) antibodies. (C) fzp21 mutant clones obtained by crossing elavC155,hsFLP,w*;UAS-mCD8::GFP,UAS-lacZ/CyO;tubP-GAL80,FRT2A/TM6,Tb,Hu or hsFlp, UAS-CD8-GFP;; FRT2A, tubGal80/TM3; OK107 with yw Flp122; sp/CyO;fz p21,ri,FRT2A/TM2. fz mutant cells show normal axon projections, similar to their wild-type counterparts. (D) fz2C1 mutant clones obtained by crossing virgins elavC155,hsFLP,w*;UAS-mCD8::GFP.,UAS-lacZ./CyO;tubP-GAL80,FRT2A/TM6,Tb,Hu or hsFlp, UAS-CD8-GFP; FRT2A, tubGal80/TM3; OK107 with males fz2C1 ri, FRT2A/TM3, Sb. Single-cell clones do not show any difference in their projection pattern compared to wild-type cells. (E) fzH51, fz2C1 double mutant clones obtained by crossing elavC155,hsFLP,w*;UAS-mCD8::GFP.,UAS-lacZ./CyO;tubP-GAL80,FRT2A/TM6,Tb,Hu with yw,hsflip; ;fz H51fz2 C1ri FRT2A/TM2. Loss of both fz and fz2 does not influence β-lobe growth, thus excluding possible compensatory effects. (F) Appl expression in third instar larvae eye disc. (G–I) Tangential adult eye sections in areas around the equator. The colored bars indicate the orientation of the ommatidia. (H) *dsh^1^* mutant flies show PCP defects and reduction of symmetric ommatidia. (I) *Appl* ^−/−^ adult flies show ommatidia orientation comparable to wild-type flies (G). (J) APPL and Vang localization during development in brain of flies expressing a EYPF tagged form of Vang under the control of Actin promoter. Immuno-fluorescence analysis using anti-APP-Cterm (blue), anti-GFP (green), and anti-FasII (red) antibodies. The images are single confocal stacks (scale bar, 15 µm). APPL and Vang are expressed in mutually exclusive compartments in the developing retina.
(PDF)

Figure S6 APP proteins are found in core PCP complexes. (A) APP expression levels in wild-type MEFs, KO MEFs, or KO MEFs stably transfected with APP. Two clones of rescue MEFs were analyzed. Clone B shows detectable APP levels and was used for the Wnt5 stimulation assay. (B) Co-immunoprecipitation (Co-IP) of Appl-FLAG and Vang-Myc. The tagged proteins were co-expressed in HEK293T cells and immunoprecipitated with anti-Myc antibody. Appl-FLAG can be precipitated upon IP of Vang. The Co-IP in this direction is weaker than after pull-down of Appl-Flag. (C) Co-IP of Appl-FLAG and human Vangl2-HA. Appl-FLAG can be precipitated upon IP of human Vangl2-HA. (D) Co-IP of APP (C99)-FLAG and Vangl2-HA. The tagged proteins were immunoprecipitated from whole cells with anti-HA antibody. APP (C99)-FLAG can be precipitated upon IP of human Vangl2-HA. (E) Control co-immunoprecipitation of overexpressed Appl-FLAG and Vangl2-HA. The proteins were separately expressed in different cells plated in two different dishes and pooled during the Co-IP procedure. The IP was performed with anti-FLAG and anti-HA antibody and followed by Western blot analysis. The proteins do not co-immunoprecipitate when expressed in different populations of cells. (F) Co-immunoprecipitation (Co-IP) of Appl-FLAG and Fz-GFP. Appl-FLAG can be precipitated upon IP of Fz.
(PDF)

## Abbreviations

Abl: Abelson kinase
AD: Alzheimer's disease
APP: Amyloid precursor protein
APP-C99: C-terminus of human APP
APPL: APP Like
ApplΔC: APPL delta C-terminal domain
dKO: double knock-out
Dsh: *Drosophila* Dishevelled
Dvl: mammalian Dishevelled
Dvl2: Dishevelled2
FasII: FascilinII
Fmi: Flamingo
Fz: Frizzled
Fzd5: Frizzled 5
MB: Mushroom Bodies
MEF: mouse embryonic fibroblast
PCP: Wnt Planar Cell Polarity
sAPPL: soluble APPL
Vangl2: Van Gogh2
Vang/Stbm: Van Gogh/Strabismus
