# Supplementary material for: The Drosophila Homologue of the Amyloid Precursor Protein Is a Conserved Modulator of Wnt PCP Signaling
Source: PLoS Biol. 2013 May 14;11(5):e1001562. doi: 10.1371/journal.pbio.1001562 (PMC3653798; doi:10.1371/journal.pbio.1001562)
Supplement: Figure S1 — Appl is required during the development of Mushroom Bodies for α- and β-lobe growth. (A) The table shows the number of brains analyzed to characterize the Appl loss of function phenotype. (PDF) [file pbio.1001562.s001.pdf]

A

Genotype	n	α loss	β loss
Appl-/-	101	14%	12%
Appl-/+	97	0	0
