# Supplementary material for: The Drosophila Homologue of the Amyloid Precursor Protein Is a Conserved Modulator of Wnt PCP Signaling
Source: PLoS Biol. 2013 May 14;11(5):e1001562. doi: 10.1371/journal.pbio.1001562 (PMC3653798; doi:10.1371/journal.pbio.1001562)

A

Genotype	n	α loss	β loss
Appl -/-	101	14%	12%
Appl +/-	97	0	0
Control clones	41	0	0
Appl -/- clones	44	0	10%

B

Genotype	n	β loss
Appl -/-	101	12%
Appl -/+	97	0
Appl -/-, rescue fl -APPL	45	2%
Appl -/+ , rescue fl -APPL	35	0
Appl -/-, rescue sAPPL	50	12%
Appl -/+, rescue sAPPL	23	0
Appl -/-, rescue APPL ΔC	54	11%
Appl -/+, rescue APPLΔC	47	0
Appl -/-, rescue control (driver)	47	13%

C

Genotype	n	α loss	β loss
Appl -/-	101	14%	12%
Appl -/+	97	0	0
Appl -/- , rescue sAPPL	50	4%	12%
Appl -/+, rescue sAPPL	23	0	0
Appl -/- , rescue control (driver)	47	13%	13%

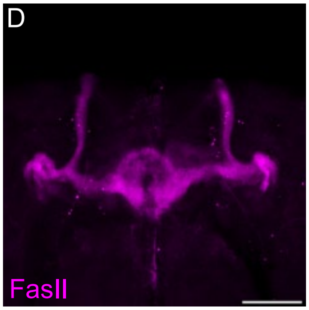

Supplement: Figure S2 — APPL is required cell-autonomously for the β-lobe outgrowth. (A)The table lists the number of brains analyzed in the MARCM experiments. (B, C) The table lists the number of brains analyzed in the rescue experiments. (D) Adult MB lobes labeled with FascilinII II antibody (FasII). The image is a z-projection of confocal image stacks (scale bar, 50 µm). Morphologically normal αβ neurons in Appldw*/Y;UAS-sAppl/+;P247Gal4 adult brains. The reintroduction of soluble APPL in MBs during development strongly reduces the loss of the α lobe. (PDF) [file pbio.1001562.s002.pdf]
