# Supplementary figures and images for: The Drosophila Homologue of the Amyloid Precursor Protein Is a Conserved Modulator of Wnt PCP Signaling
Source: PLoS Biol. 2013 May 14;11(5):e1001562. doi: 10.1371/journal.pbio.1001562 (PMC3653798; doi:10.1371/journal.pbio.1001562)

Appl  $-/-$ , Abl  $-/+$

48 APF

A

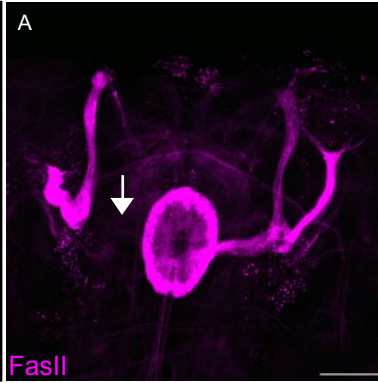

FasII

Supplement: Figure S3 — Abelson kinase is the downstream effector of APPL in MB development. (A) Structure of α/β neurons of a Appld;;Abl4 48APF brain. The image is a z-projection of confocal image stacks (scale bar, 50 µm). The MBs are labeled with anti-FasII antibody. β-lobe loss is detectable already at 48 APF, similarly to what is observed in the Appl −/− background. (PDF) [file pbio.1001562.s003.pdf]

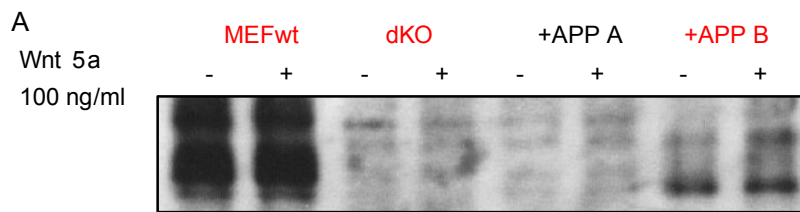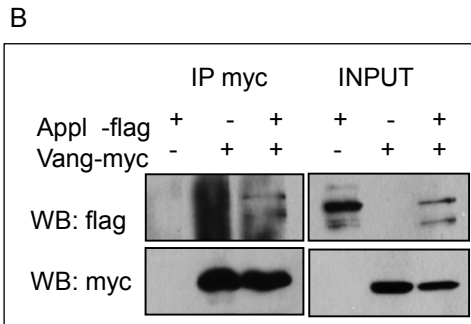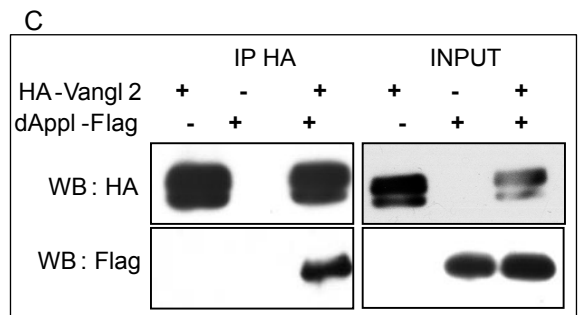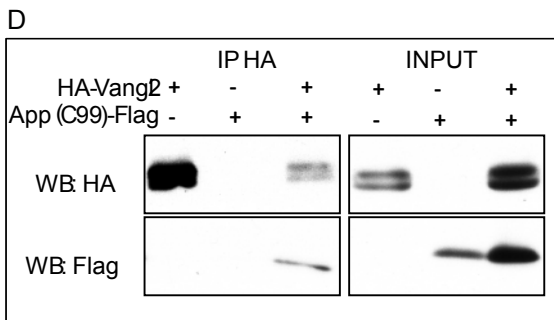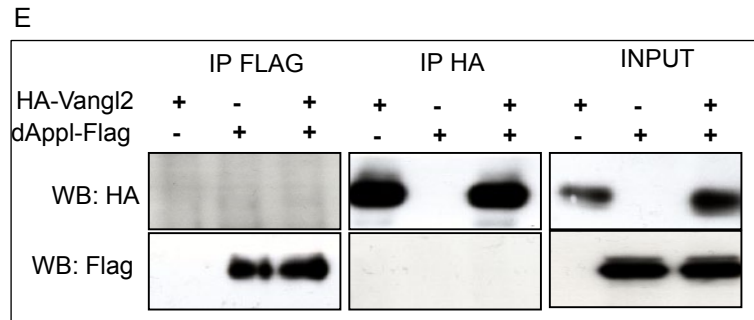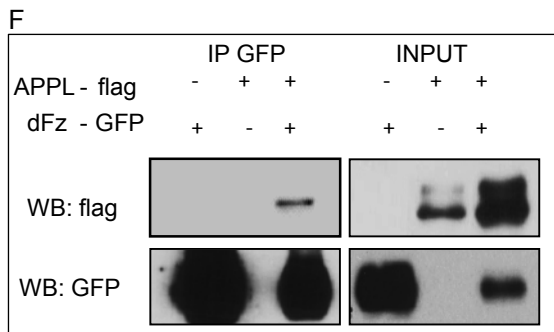

Supplement: Figure S6 — APP proteins are found in core PCP complexes. (A) APP expression levels in wild-type MEFs, KO MEFs, or KO MEFs stably transfected with APP. Two clones of rescue MEFs were analyzed. Clone B shows detectable APP levels and was used for the Wnt5 stimulation assay. (B) Co-immunoprecipitation (Co-IP) of Appl-FLAG and Vang-Myc. The tagged proteins were co-expressed in HEK293T cells and immunoprecipitated with anti-Myc antibody. Appl-FLAG can be precipitated upon IP of Vang. The Co-IP in this direction is weaker than after pull-down of Appl-Flag. (C) Co-IP of Appl-FLAG and human Vangl2-HA. Appl-FLAG can be precipitated upon IP of human Vangl2-HA. (D) Co-IP of APP (C99)-FLAG and Vangl2-HA. The tagged proteins were immunoprecipitated from whole cells with anti-HA antibody. APP (C99)-FLAG can be precipitated upon IP of human Vangl2-HA. (E) Control co-immunoprecipitation of overexpressed Appl-FLAG and Vangl2-HA. The proteins were separately expressed in different cells plated in two different dishes and pooled during the Co-IP procedure. The IP was performed with anti-FLAG and anti-HA antibody and followed by Western blot analysis. The proteins do not co-immunoprecipitate when expressed in different populations of cells. (F) Co-immunoprecipitation (Co-IP) of Appl-FLAG and Fz-GFP. Appl-FLAG can be precipitated upon IP of Fz. (PDF) [file pbio.1001562.s006.pdf]
