# Supplementary material for: The Drosophila Homologue of the Amyloid Precursor Protein Is a Conserved Modulator of Wnt PCP Signaling
Source: PLoS Biol. 2013 May 14;11(5):e1001562. doi: 10.1371/journal.pbio.1001562 (PMC3653798; doi:10.1371/journal.pbio.1001562)
Supplement: Figure S4 — Dsh phosphorylation is required for MB development. (A) The table lists the number of brains analyzed in the dsh rescue experiments. (PDF) [file pbio.1001562.s004.pdf]

**Genotype**	**n**	**β loss**
Dsh^1	36	30%
Dsh^1, Dsh>Dsh-GFP	31	0
Dsh^1, Dsh>Dsh -Y473F-GFP	41	29%
