# Supplementary material for: The Drosophila Homologue of the Amyloid Precursor Protein Is a Conserved Modulator of Wnt PCP Signaling
Source: PLoS Biol. 2013 May 14;11(5):e1001562. doi: 10.1371/journal.pbio.1001562 (PMC3653798; doi:10.1371/journal.pbio.1001562)

A

Genotype	n	β loss
Appl -/-	101	12%
Appl +/-	97	0
dsh[1]	36	30%
Appl -/-, fz -/+	28	21%
Appl -/+, fz -/+	21	0
Appl -/-, fzDN	28	18%
fzDN	19	0
Appl -/-, Vang +/-	21	33%
fz clones	40	0

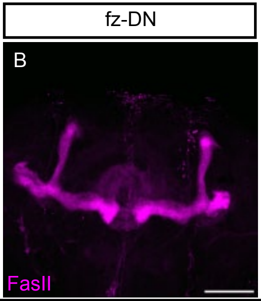

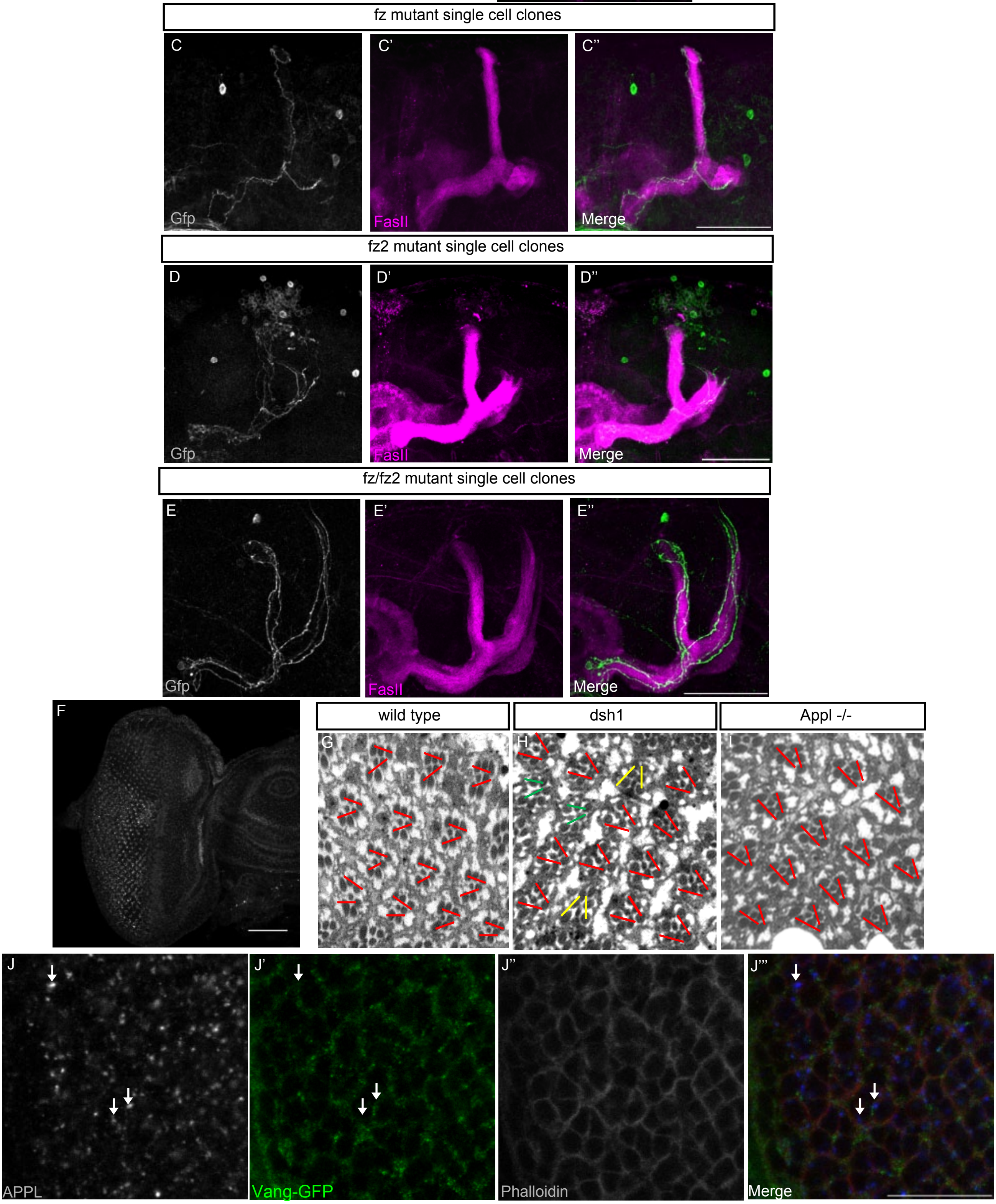

Supplement: Figure S5 — Appl interacts with the PCP signaling during MB development. (A) The table lists the number of brains analyzed in the PCP genetic interaction experiments. (B) Structure of α/β neurons labeled with anti-FasII antibody. The image is a z-projection of confocal image stacks (scale bar, 50 µm). Morphologically normal α/β neurons in UAS-FzDN/+;P247Gal4/+ adult brains. Expression of a dominant negative form of fz in the MB is not sufficient to induce defects. (C–E) Z-projections of confocal image stacks of GFP-labeled MARCM clones. Immuno-fluorescence analysis of adult MB, using anti-GFP (green) and anti-FasII (magenta) antibodies. (C) fzp21 mutant clones obtained by crossing elavC155,hsFLP,w*;UAS-mCD8::GFP,UAS-lacZ/CyO;tubP-GAL80,FRT2A/TM6,Tb,Hu or hsFlp, UAS-CD8-GFP;; FRT2A, tubGal80/TM3; OK107 with yw Flp122; sp/CyO;fz p21,ri,FRT2A/TM2. fz mutant cells show normal axon projections, similar to their wild-type counterparts. (D) fz2C1 mutant clones obtained by crossing virgins elavC155,hsFLP,w*;UAS-mCD8::GFP.,UAS-lacZ./CyO;tubP-GAL80,FRT2A/TM6,Tb,Hu or hsFlp, UAS-CD8-GFP; FRT2A, tubGal80/TM3; OK107 with males fz2C1 ri, FRT2A/TM3, Sb. Single-cell clones do not show any difference in their projection pattern compared to wild-type cells. (E) fzH51, fz2C1 double mutant clones obtained by crossing elavC155,hsFLP,w*;UAS-mCD8::GFP.,UAS-lacZ./CyO;tubP-GAL80,FRT2A/TM6,Tb,Hu with yw,hsflip; ;fz H51fz2 C1ri FRT2A/TM2. Loss of both fz and fz2 does not influence β-lobe growth, thus excluding possible compensatory effects. (F) Appl expression in third instar larvae eye disc. (G–I) Tangential adult eye sections in areas around the equator. The colored bars indicate the orientation of the ommatidia. (H) dsh1 mutant flies show PCP defects and reduction of symmetric ommatidia. (I) Appl −/− adult flies show ommatidia orientation comparable to wild-type flies (G). (J) APPL and Vang localization during development in brain of flies expressing a EYPF tagged form of Vang under the control of Act [file pbio.1001562.s005.pdf]
